# A Method for Lung Boundary Correction Using Split Bregman Method and Geometric Active Contour Model

**DOI:** 10.1155/2015/789485

**Published:** 2015-05-18

**Authors:** Changli Feng, Jianxun Zhang, Rui Liang

**Affiliations:** ^1^Department of Information Science and Technology, Taishan University, Taian 271021, China; ^2^Tianjin Key Laboratory of Intelligent Robotics, Institute of Robotics and Automatic Information System, College of Computer and Control Engineering, Nankai University, No. 94 Weijin Road, Tianjin 300071, China

## Abstract

In order to get the extracted lung region from CT images more accurately, a model that contains lung region extraction and edge boundary correction is proposed. Firstly, a new edge detection function is presented with the help of the classic structure tensor theory. Secondly, the initial lung mask is automatically extracted by an improved active contour model which combines the global intensity information, local intensity information, the new edge information, and an adaptive weight. It is worth noting that the objective function of the improved model is converted to a convex model, which makes the proposed model get the global minimum. Then, the central airway was excluded according to the spatial context messages and the position relationship between every segmented region and the rib. Thirdly, a mesh and the fractal theory are used to detect the boundary that surrounds the juxtapleural nodule. Finally, the geometric active contour model is employed to correct the detected boundary and reinclude juxtapleural nodules. We also evaluated the performance of the proposed segmentation and correction model by comparing with their popular counterparts. Efficient computing capability and robustness property prove that our model can correct the lung boundary reliably and reproducibly.

## 1. Introduction

X-ray computed tomography (CT) is the most sensitive way for lung nodules detection [1], and detecting lung nodules using CT has become an increasingly important issue [2]. In this task, the lung region segmentation is a critical first step which can minimize the analytical region and system computation. Furthermore, the automated lung segmentation method is also needed due to the large number of axial slices that are produced by the multidetector-row CT scanner [3]. In addition, because juxtapleural nodules are contiguous with the chest wall and they have similar density to lung region, those nodules are always incorrectly excluded from the lung region. Thus the method, which can extract the lung region without the loss of any juxtapleural nodules from CT slices, should not only include the lung segmentation process, but also correct the defective boundary that surrounds the juxtapleural nodule.

Several methods have been proposed for segmenting lung region from CT slice images. The most used techniques are the intensity-based methods [4–7], which are based on the intensity difference between the lung tissue and other tissues. In this kind of models, to separate the whole region into two parts, a fixed threshold value is selected. Although this method is simple and fast to implement, it has an inherent limit that there is not an ideal fixed threshold that works well for all the images. Thus, the optimal threshold [8] is proposed, which gives an adaptive threshold for every slice image. But this model is also based on the theory of the fixed threshold method which makes the model lack the ability of segmenting the CT images that contain inhomogeneous intensity.

As another type of segmentation technique, the active contour model is also introduced to obtain a more accurate segmented result in the chest CT images. In [9–12], the proposed active contour models combine the gradient information or the curvature information which makes the level set function stop on the boundary with high gradient or curvature values. However this kind of model does not easily converge to boundary concavity, which leads to a rough boundary [13]. As another type of information, the regional information is also used in active contour models [14–19]. Chan-Vese [14] proposes the well-known CV model which can segment the intensity homogeneous images quickly and robustly. Li [15] presents a local binary fitting (LBF) model which can segment the intensity inhomogeneous images successfully. Yang [20] combines the CV model, the LBF model, the global convex segmentation method, and the split Bregman method together to construct a new active model that is a global convex and can get the global minimum no matter where the initial contour lies.

As for the edge correction methods, the existing methods can be subdivided into two families: the morphology-based methods [5, 7, 21–24] and the geometry-feature-based models [25, 26]. The commonly used rolling-ball methods [7, 21, 22], which drive a ball along the edge contour to reinclude juxtapleural nodules, belong to the morphology-based methods. In [5, 23, 24], sequences of erosion and dilation operations are combined to smooth the segmented lung boundary; they belong to the morphology-based methods too. Those morphology-based methods are easy and fast to implement, but the correction accuracy hugely depends on the radii of the morphology template. What is more, there are no proper radii that work well all the time. On the other side, the curvature information of the lung contour is used to identify the juxtapleural nodules [25, 26], but the curvature information is not a robust criterion for its sensitive properties to the rapid curvature changes and the noise. Yim and Hong [27] propose a contour tracing method to smooth the 2D contour; Pu et al. [28] present an adaptive border marching (ABM) method to reinclude all the juxtapleural nodules; Yim et al. [2] correct the boundary by evolving the initial identified boundary with a defined scope and finding the optimal boundary. However, all those methods depend on the initial identified boundary greatly. If the initial boundary is recognized incorrectly, the correction method effect may be worse.

In this paper, a new lung extract method without the loss of the juxtapleural nodules is proposed by means of the CV model, the LBF model, the innovative edge detection function, the split Bregman method, and the geometric active contour model. First of all, a new edge detection function is defined with the help of the classical structure tensor theory. Then a new active contour model, which combines the global information, the local information, and the new edge information, is proposed. Besides, the proposed active contour model is converted to a convex segmentation model and is solved by the split Bregman method. After that, the central airway is excluded automatically. In the next step, the fractal theory is applied to detect the defective edge that is caused by juxtapleural nodules and the blood vessel and the airway. Finally, the geometric active contour model is introduced to correct that detected defective boundary.

The organization of the paper is as follows. The new edge detection function, the segmentation active contour model, the correction geometric method, and other processes are discussed in Section 2. Numeric experiments are given in Section 3. The conclusion of the paper is offered in Section 4.

## 2. Methods

**Figure 1:**
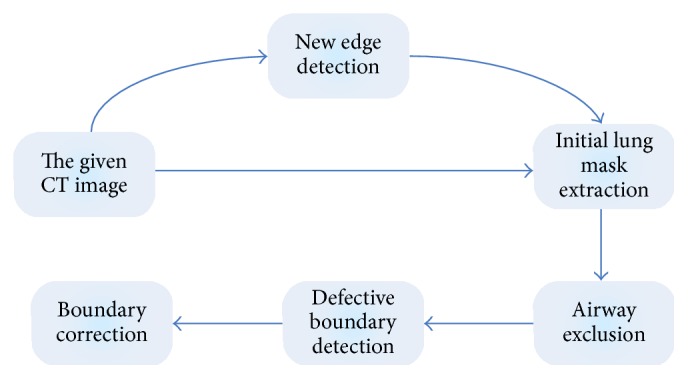
The schemes of the proposed lung correction method.

As shown in Figure 1, the proposed model consists of three major steps. Firstly, the lung initial mask is extracted by an active contour model. Secondly, according to the airway position in the preslice CT image and the location relationship between human organs and the rib tissue, the central airway in every slice of the CT scan is excluded automatically. Then, the lung region is divided into several smaller blocks using a grid line. Thirdly the fractal theory is used to detect the lung boundary that is contiguous to juxtapleural nodules. Finally, the geometric active contour model is introduced to correct the defective lung boundary that is caused by juxtapleural nodules.

### 2.1. The New Edge Detection Function

As a traditional edge detection function, *g* is defined as follows:

(1) $$\begin{array}{c} g\left(I\right)=\frac{1}{1+{\left|\nabla \left(G_{\sigma }*I\right)\right|}^{2}}, \end{array}$$

where *G*_*σ*_ is a Gaussian kernel function with the standard deviation of *σ*. From (1), it is easy to find that *g* is sensitive to different noise sources and weak boundaries. So when *g* is used, it may make the level set evolution unstable if the detected edge is affected by different noise sources or weak boundaries.

In order to overcome the disadvantage, the classical structure tensor theory [29] is used to construct a new edge detection function. For a scalar image **I**, the classical structure tensor *J*_*σ*_ is defined as follows:

(2) $$\begin{array}{c} J_{\sigma }=\left(\begin{array}{cc} K_{\sigma }*I_{x}^{2} & K_{\sigma }*I_{x}I_{y}\\ K_{\sigma }*I_{x}I_{y} & K_{\sigma }*I_{y}^{2} \end{array}\right), \end{array}$$

where *K*_*σ*_ is a Gaussian kernel function of size 3 × 3 with the standard deviation *σ* and *I*_*x*_ and *I*_*y*_ denote the partial derivatives of the given image.

To be expressed simply, *J*_*σ*_ is written as $\left(\begin{array}{cc} J_{xx} & J_{xy}\\ J_{xy} & J_{yy} \end{array}\right)$. Note that the matrix *J*_*σ*_ is positive semidefinite; its eigenvalues are labeled as *ρ*_1_, *ρ*_2_, *ρ*_1_ ≥ *ρ*_2_. What is more, it is easy to find that *ρ*_1_ ≈ *ρ*_2_ ≈ 0 in the flat region, *ρ*_1_ ≫ *ρ*_2_ ≈ 0 when the point is on the straight line edge, and *ρ*_1_ ≫ *ρ*_2_ ≫ 0 when it is a corner point. That is, the difference Δ*ρ* between the two eigenvalues gets a small value in the flat region and a big value on the edge of the image, respectively.

Thus we can select a threshold to distinguish those two regions from each other; let *S*_*T*_ = {*x*∣*x* ∈ *T*}, *T* = *ρ*_1_ − *ρ*_2_; then the threshold is defined as follows:

(3) $$\begin{array}{c} T_{c}=\overset{-}{T}+\kappa \sigma_{T}, \end{array}$$

where $\overset{-}{T}$ and *σ*_*T*_ are the mean and the standard deviation of *S*_*T*_, respectively, and *κ* is a constant-weight that controls the difference between *T*_*c*_ and $\overset{-}{T}$.

By virtue of *T*_*c*_, a new edge detection function is calculated as follows:

(4) $$\begin{array}{c} g^{*}\left(x,y\right)=\left\{\begin{array}{cc} 0, & T\left(x,y\right)\leq T_{c}\\ 1, & T\left(x,y\right)>T_{c}. \end{array}\right. \end{array}$$

**Figure 2:**
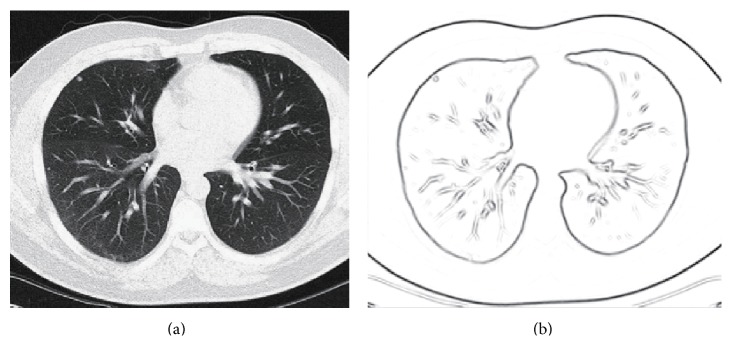
The edge detection result of *g* ^∗^. (a) The original image; (b) the detected edge.

In Figure 2(b), there is the detected edge of *g* ^∗^, from which it is easy to find that *g* ^∗^ gives a perfect edge of the image in Figure 2(a). Furthermore, this result is very similar to the observation of human. What is important is that, except for offering of the edge information to the proposed active contour model, *g* ^∗^ can also serve as an adaptive weight of the proposed model, which is shown in the following subsection.

### 2.2. Initial Lung Mask Extraction

Due to the partial volume effect (PVE) or the similar density between the juxtapleural nodules and the chest wall, CT images often have inhomogeneous intensity around the lung boundary. However, the most of existing methods cannot segment those CT images accurately. So, we propose a new combined model to segment this kind of CT images. The new model is a combination of the global-region-information and local-region-information with an adaptive weight. Besides, we convert the new active contour model to a convex minimization model which can ensure the robustness and efficiency of segmentation result.

As mentioned above, CV model and LBF model are active contour models (ACM) that are based on global intensity information and local region intensity information correspondingly; thus those two models are integrated into the new model dynamically to inherit their advantages. Its energy function is as follows:

(5) $$\begin{array}{c} E^{\text{GLg}}=\alpha E^{\text{CV}}+\beta E^{\text{LBF}}+\gamma E_{g^{*}}^{L}+\nu E^{R}, \end{array}$$

where *α*, *β*, *γ*, and *ν* are four positive constants which control the energy ratio of the CV model, the LBF model, the length term, and the regularization term correspondingly in the proposed energy function; *E*_*g*^∗^_^*L*^ and *E* ^*R*^ are the length term and the regularization term correspondingly; *E* ^CV^ and *E* ^LBF^ are the energy function of CV model and LBF model, which can be calculated by

(6) ECV=∑i=12ηiMiϕxIx−ci2dx,ELBF=∑i=12ξi∫∫Kωx−yIy−fix2MiϕydydxEg∗L=∫Ωg∗xδεϕx∇ϕxdxER=∫Ω∇ϕx−12dx,

where *η*_1_, *η*_2_, *ξ*_1_, and *ξ*_2_ are four positive constants that balance the intensity-based energy in the inner and outer of the curve *C* and *K*_*ω*_ denotes a Gaussian kernel function with standard deviation of *ω* and window size of (4*ω* + 1)×(4*ω* + 1). The rest of the terms are defined as follows:

(7) $$\begin{array}{c} H_{\varepsilon }\left(x\right)=\frac{1}{2}\left[1+\frac{\pi }{2}\arctan\left(\frac{x}{\varepsilon }\right)\right]\\ \delta_{\varepsilon }\left(x\right)=\frac{1}{\pi }\frac{\varepsilon }{\varepsilon^{2}+x^{2}}\\ M_{1}=H_{\varepsilon }\left(x\right),\;\;M_{2}=1-M_{1}\\ c_{i}=\frac{\int_{\Omega }M_{i}\left(\phi \left(x\right)\right)I\left(x\right)dx}{\int_{\Omega }M_{i}\left(\phi \left(x\right)\right)dx},\;i=1,2\\ f_{i}=\frac{K_{\omega }*\left(M_{i}\left(\phi \left(x\right)\right)I\left(x\right)\right)}{K_{\omega }*M_{i}\left(\phi \left(x\right)\right)},\;i=1,2\\ \alpha =\alpha^{\prime }\cdot g^{*},\;\;\beta =\beta^{\prime }\left(1-g^{*}\right), \end{array}$$

where *α*′ and *β*′ are two positive constants that denote the adaptive weights of the first two terms in (5).

It is worth noting that the proposed *g* ^∗^ has two roles in (5). One is the factor of the length term which can drive the evolving level set function toward the detected *g* ^∗^. By this term the computational efficiency will be improved. The other role is the adaptive weight, which can assign appropriate combined energy to every point. With the help of this term, the edge point will be given more local information which helps to determine the precise boundary. It is the major improvement of our proposed method which makes our proposed model outperform their *g*-based counterparts [20].

The energy function (5) can be minimized by solving the following gradient flow:

(8) ∂ϕ∂t=αδεϕ−η1I−c12+η2I−c22 +βδεϕ−ξ1e1+ξ2e2+ν∇2ϕ−div∇ϕ∇ϕ +γδεϕdivg∗∇ϕ∇ϕ,

where *e*_*i*_(*x*) = ∫*K*_*ω*_(*y* − *x*) | *I*(*x*) − *f*_*i*_(*y*)|^2^ *dy*.

As most active contour models, new model (5) is also prone to the local minimum. As mentioned above, LCV model is prone to the local minimum. To overcome this defect, (5) is converted to a convex minimization problem; then it is solved by the split Bregman method. First of all, in general, let *ξ*_1_ = *ξ*_2_ = *η*_1_ = *η*_2_ = 1; then (8) is written as follows:

(9) ∂ϕ∂t=γδεϕdivg∗∇ϕ∇ϕ+μδεϕF1+F2 +ν∇2ϕ−div∇ϕ∇ϕ,

where

(10) F1=α′g∗−I−c12+I−c22F2=β′1−g∗−e1+e2.

Note that (9) is nonconvex too; however, it can be transformed into a convex one by the globally convex segmentation (GCS) method [30]. To apply this method, the third term in (9) should be dropped. In fact, according to [20], the regularization term is used to eliminate the reinitialization process and maintain the level set function as an approximate signed distance function near the zero level set. At the same time, this term is not contained in the classical region-based models, such as the famous CV models. With only the data fitting term and the arc length term, these models work well. Actually, the reinitialization process is not encouraged for most experiments since it may cause some subtle side effects, such as preventing the detection of interior boundaries within an object, as pointed out in CV model [14]. Furthermore, in split Bregman method, we restrict *ϕ* to the interval [0,1]. In this way, the level set function *ϕ* will not blow up to very large values on both sides of the zero level set and will not cause inaccurate computation or erroneous segmentation results. Thus dropping this term is reasonable and will not affect the segmentation results of the model.

Let *γ* = 1; then we get the new gradient flow as follows:

(11) $$\begin{array}{c} \frac{\partial \phi }{\partial t}=\delta_{\varepsilon }\left(\phi \right)\left(\mathrm{div}\left(g^{*}\frac{\nabla \phi }{\left|\nabla \phi \right|}\right)+\mu \left(F_{1}+F_{2}\right)\right). \end{array}$$

With the help of the GCS method, we drop *δ*_*ε*_(**ϕ**) to get a simplified gradient flow. It is worth noting that, through *δ*_*ε*_(**ϕ**) which is dropped, the optimality solution of the simplified gradient flow is also equivalent to its original form [20]. The simplified form is defined as follows:

(12) $$\begin{array}{c} \frac{\partial \phi }{\partial t}=\left[\mathrm{div}\left(g^{*}\frac{\nabla \phi }{\left|\nabla \phi \right|}\right)+\mu \left(F_{1}+F_{2}\right)\right]. \end{array}$$

Based on (12), a new energy function can be constructed as follows:

(13) $$\begin{array}{c} E^{\text{GLg}}\left(\phi \right)=\int g^{*}\left|\nabla \phi \left(x\right)\right|dx+\int \mu \phi \left(x\right)s\left(x\right)dx, \end{array}$$

where *s*(**x**) = −(*F*_1_(**x**) + *F*_2_(**x**)).

It is interesting to find that (13) has the same solution as (11). Thus the purpose becomes finding the minimum of (13).

To guarantee model (13) gets the unique global minimum, the range of **ϕ** is restricted within a bounded interval. In this paper, the interval is [0,1]. Thus (13) can be written as follows:

(14) min⁡ϕ∈0,1EGLgϕ=min⁡ϕ∈0,1∫g∗Ix∇ϕxdx +∫μϕxsxdx.

Generally, (14) can be expressed as

(15) $$\begin{array}{c} \underset{\phi \in \left[0,1\right]}{\min}E^{\text{GLg}}\left(\phi \right)=\underset{\phi \in \left[0,1\right]}{\min}\left[{\left|\nabla \phi \right|}_{g^{*}}+\mu \langle \phi ,s\rangle \right], \end{array}$$

where

(16) $$\begin{array}{c} {\left|\nabla \phi \right|}_{g^{*}}=\int g^{*}\left(I\left(x\right)\right)\left|\nabla \phi \left(x\right)\right|dx,\\ \langle \phi ,s\rangle =\int \phi \left(x\right)s\left(x\right)dx. \end{array}$$

In order to apply the split Bregman method, we present a new term $\vec{d}$ and a penalty term that can ensure $\vec{d}$ approximate to |∇**ϕ**(**x**)| in the iteration. By those two terms, (14) is reformatted into the following form:

(17) $$\begin{array}{c} \underset{\phi \in \left[0,1\right]}{\min}E^{\text{GLg}}\left(\phi \right)=\underset{\phi \in \left[0,1\right]}{\min}\left[{\left|\vec{d}\right|}_{g^{*}}+\mu \langle \phi ,s\rangle +\frac{\lambda }{2}{\left\|\vec{d}-\nabla \phi \right\|}^{2}\right], \end{array}$$

where *λ* is a positive constant parameter.

Then the Bregman iteration is used to meet the condition $\vec{d}=|\nabla \phi (x)|$ [31], so we get the following optimization problem:

(18) ϕk+1,d→k+1=argmin⁡ϕ∈0,1,d→d→g∗+μϕ,s+λ2d→−∇ϕ−d→k2 +λ2d→−∇ϕ−d→k2,

where ${\vec{b}}^{k+1}$ is got by the Bregman iteration:

(19) $$\begin{array}{c} {\vec{b}}^{k+1}={\vec{b}}^{k}+\left(\nabla \phi^{k+1}-{\vec{d}}^{k+1}\right). \end{array}$$

The optimization solution of **ϕ** ^*k*+1^ is obtained by the optimization condition:

(20) $$\begin{array}{c} \Delta \phi =\frac{\mu \cdot s}{\lambda }+\nabla \left(\vec{d}-\vec{b}\right),\;\phi \in \left[0,1\right]. \end{array}$$

For (20), we get the approximated solution of *ϕ* by means of the Gauss-Seidel method [31], which is computed by

(21) αi,j=di−1,jx,k−di,jx,k+di,j−1y,k−di,jy,k −bi−1,jx,k+bi,jx,k−bi,j−1y,k+bi,jy,k,βi,j=14λ·sμϕi−1,j+ϕi+1,j+ϕi,j−1=14vv+ϕi,j+1−λ·sμ+αi,j,ϕi,j=max⁡min⁡βi,j,1,0.

Finally, the solution of ${\vec{d}}^{k+1}$ is acquired by the Shrink process [31]:

(22) d→k+1=shrinkg∗b→k+∇ϕk+1,1λ=shrinkb→k+∇ϕk+1,g∗λ,

where the Shrink process is defined as follows:

(23) $$\begin{array}{c} \text{shrink}\left(x,\theta \right)=\left\{\begin{array}{cc} \frac{x}{\left|x\right|}\max\left(\left|x\right|-\theta ,0\right), & x\neq 0\\ 0, & x=0. \end{array}\right. \end{array}$$

**Figure 3:**
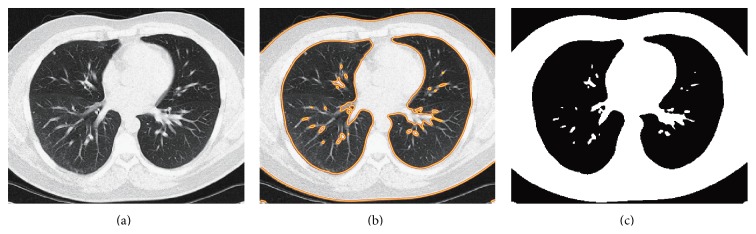
The segmentation result and the obtained initial mask. (a) The original image; (b) the segmentation result; (c) the obtained initial lung mask.

As shown in Figure 3, for the given images **I**, update *s* in (21) by calculating the mean intensity in the regions **ϕ** > 0 and **ϕ** < 0. Then update **ϕ** according to (21) until its convergence is achieved. By this way, the final segmentation result is obtained by the boundary of the following set:

(24) $$\begin{array}{c} \left\{x\in \Omega \;|\;\phi^{\text{final}}\left(x\right)>0.5\right\}. \end{array}$$

The segmentation result of the given image in Figure 3(a) is shown in Figure 3(b), and the binary image of the segmentation result is displayed in Figure 3(c).

### 2.3. Airway Exclusion

Because the inner of airway has the approximate intensity with the lung region, it is often included in the final segmentation result of the intensity-information-based image segmentation model. However, this part of tissue, especially the central airway, is useless for lung nodules detection; thus it needs to be excluded from the above segmentation result.

In fact, it is easy to find that the trachea and bronchi out of the lung region are faraway from the ribs and there is less osseous tissue in its neighborhood, while the outer boundary of the lung region is contiguous with the rib. Thus, for every subregion of the segmentation result, a morphology dilation process is applied to detect whether there is an osseous tissue in the dilated region. If none of the osseous tissues exists, this subregion is excluded from the lung region of the current CT slice. Until all the slices in the CT scan are processed, the majority of those regions are removed already.

However, the performance of the exclusion process is not always perfect all the time. For some slices of some patients, the tracheal cartilages around the trachea and bronchi out of the lung region are very obvious; the dilated neighborhood region contains some tracheal cartilage that is regarded as the osseous tissue, so the inner parts of them are preserved.

**Figure 4:**
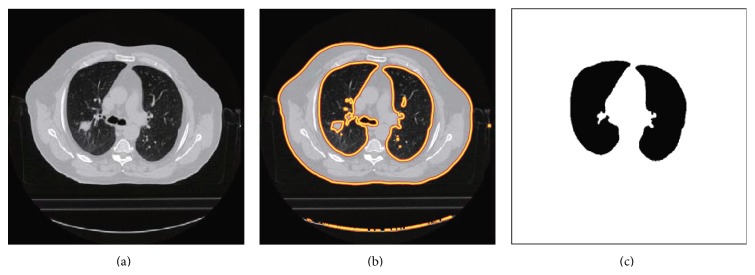
The airway-free initial lung mask. (a) The original image; (b) the segmentation result; (c) the result after extracting central airway.

In order to tackle this problem, a further process is introduced. In the CT slice, it is seen that the tracheal cartilage is obvious in some slice, but it does not always exist in the previous or successive slices for its size limit in the *z*-axis. What is more, it is interesting to observe that the inner parts of the trachea and bronchi are excluded in those tracheal cartilage vanished slices. Besides, the locations of organs are approximately similar to each other among the adjacent slices. Therefore we can use these two properties to exclude the inner region of the central airway. In this method, from the second slice of the CT scan, each subregion in the current slice is checked for whether the equivalent location in the previous slice is excluded. If the same subregion is removed, the current region will be excluded from lung region too. Until all slices are handled, the major central airway-surrounded regions have been excluded already. In addition, the consequence of subsection is also given in Figure 4.

### 2.4. Defective Boundary Detection

For juxtapleural nodules, they have the approximate intensity with the surrounding area, so they are often incorrectly excluded from the lung region. But those nodules hold a higher rate of being malignant tumors than other inner nodules, so the performance of the CAD system will be affected if those nodules are not reincluded into the lung region.

Hence, according to the fact that the contour of the lung region is smooth and the smoothness of the boundary on the segmented image is damaged by the juxtapleural nodule, a new boundary detection method is proposed to detect the defective boundary by means of the fractal theory.

First of all, the minimum enclosing rectangle (MER) of the lung region on 2D slice is acquired; then it is uniformly divided into 10 × 10 blocks by a gird line. The number of the blocks is chosen according to the actual size of the lung region and the size of lung nodules. Then the blocks that contain the boundary of the segmentation result, which is called the boundary block, are detected. In order to visualize the detection process, the result of this subsection is shown in Figure 6. Note that when there is only one lung subregion, the blocks should be set to 2 × 2 to avoid the phenomenon that the size of the block is too small to carry the operation on it.

For every boundary block, the fractal theory is adopted to account the fractal dimension of the inner boundary line. Furthermore, for easy and automatic implementation, the box-counting method is selected to compute the fractal dimension among so many techniques [32]. By this method, every boundary block is covered by a series of grids, whose sizes have a progressive decrease. For each of the girds, the following two values are recorded: the number of square boxes intersected by the image, *N*(*s*), and the side length of the squares, *s*. The regression slope *D* of the straight line formed by plotting log⁡(*N*(*s*)) against log⁡(1/*s*) is the fractal dimension of the current block which can be evaluated as follows [32]:

(25) $$\begin{array}{c} \log\left(N\left(s\right)\right)=\log\left(K\right)+D\log\left(\frac{1}{s}\right), \end{array}$$

where *K* is a constant and *N*(*s*) is proportional to (1/*s*)^−*D*^.

**Figure 5:**
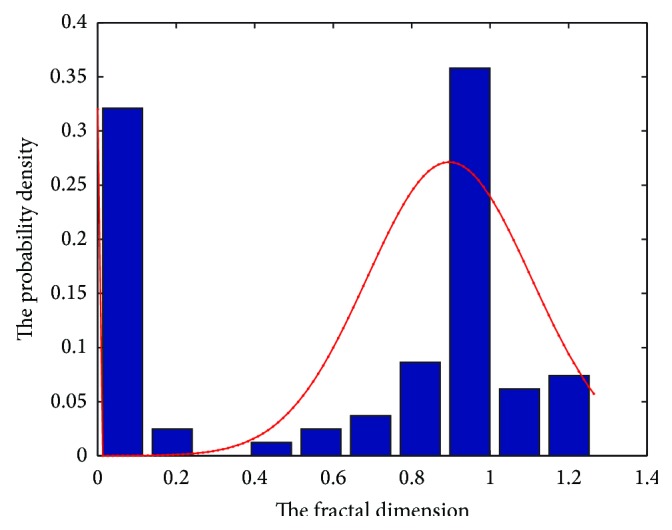
The histogram of all the fractal dimension values in all edge blocks.

As mentioned above, the normal boundary of the lung region on 2D slice is smooth, so the fractal dimension of the block that contains those kinds of boundary is smaller than that of the boundary block that contains the defective boundary. Until all the boundaries are calculated by the box-counting method, all the boundary blocks can be categorized into two classes: the blocks with a small fractal dimension that contain the normal smooth boundary and the blocks with a big value that contain the defective boundary that is caused by the juxtapleural nodule, which can be seen clearly from the histogram in Figure 5. Thus we can select an appropriate threshold to distinguish those blocks into classes.

Let *d*_*i*_ denote the fractal dimension of the *i*th boundary block; *N*_*b*_ indicates the overall number of the boundary block, *S*_*f*_ = {*d*_*i*_∣*i* = 1,2,…, *N*_*b*_}; the threshold *T*_*f*_ can be defined as follows:

(26) $$\begin{array}{c} T_{f}=\overset{-}{d}+s_{d}, \end{array}$$

where $\overset{-}{d}$ and *s*_*d*_ are the mean value and the standard deviation of *S*_*f*_, respectively. Furthermore, the threshold in (26) is given by the strategy of trial-and-error empirically. In our experiments, both the mean and the valley value of the histogram are not suitable; thus we select an optimal value after many experiments.

**Figure 6:**
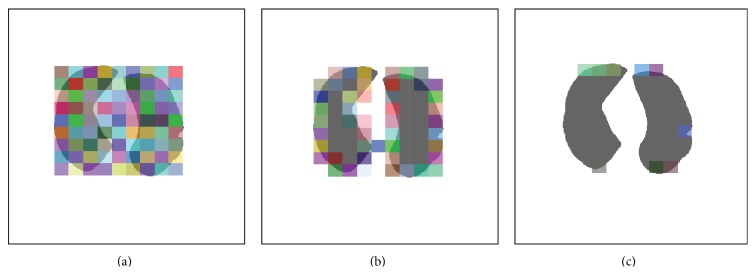
The process of detecting defective boundary block. (a) The whole block setting; (b) the boundary block; (c) the detected defective boundary block.

With the help of *T*_*f*_, the block is regarded as the block that contains the defective boundary if its fractal dimension is bigger than *T*_*f*_. Then those detected blocks will be corrected in the following subsection. As shown in Figure 6(c), the detected defective boundary by ${\overset{-}{T}}_{f}$ is very accurate, and it is nearly similar to the observation of human. Note that it is the first time that the fractal theory is used to identify the defective lung boundary that surrounds juxtapleural nodules in CT slices. Besides, the lung-inner blood vessel is also excluded by the segmentation method for their higher density. In fact, those regions should reinclude the lung region to get an accurate nodule-detection result. It is interesting that, except the defective boundary that is caused by juxtapleural nodules, our model can detect those boundaries that are caused by those blood vessels too and regard them as the defective boundary. Furthermore, previous methods only take local properties of the boundary into account, which makes the model sensitive to the robustness of those local properties. But the proposed model integrated the global boundary properties with the local boundary properties together by the statistical method. Thus the detected result will be more robust to different shapes of the lung boundary.

### 2.5. Boundary Correction Based on Geometric Deformable Models

**Figure 7:**
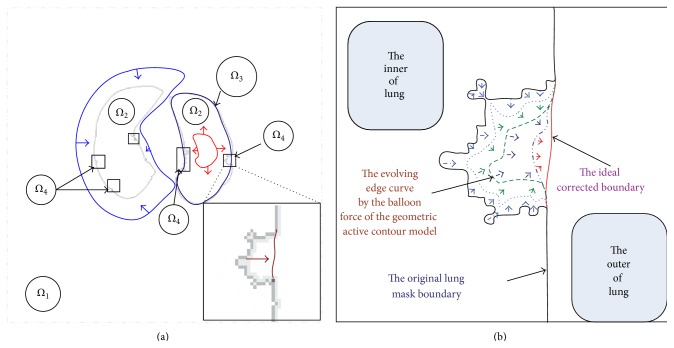
The figure of the geometric active contour model and the purpose of the proposed correction method. (a) The general description of the geometric active contour model; (b) the purpose of our method.

As shown in Figure 7(a), the whole image is divided into four parts: *Ω*_1_, *Ω*_2_, *Ω*_3_, and *Ω*_4_. *Ω*_1_ is the outside of the lung region except for those points in the defective boundary blocks, *Ω*_2_ denotes inner parts of the lung region, *Ω*_3_ indicates the set of boundary points in the correct boundary block, and the detected defective boundary block region is labeled as *Ω*_4_.

Besides, according to the purpose of the correction process and the boundary information, we also give the different curve evolving strategies for all four kinds of regions. In *Ω*_1_, if the evolving curve passes through the real boundary, the curve should be shrunk. Conversely, in *Ω*_2_, the curve will be expanded if the curve converges into the inner of the lung. For *Ω*_3_, due to those edge points on the accurate boundary already, there is no need to correct them. Therefore, if the curve is on those points, it should be kept still to avoid useless computation. Finally, for *Ω*_4_, because they contain the defective boundary, the hole should be filled while keeping the boundary smooth. Thus the curve evolution is proposed to expand the defective boundary in the hole by a balloon-force-like technique. As shown in Figure 7(b), by this way, the defective inner boundary will be expanded to the ideal position all the time.

From Figure 7, what needs to be done is expanding the whole lung region from the inner part of the lung region by a balloon-force-like power until we get an optimal smooth boundary while keeping the correct boundary still in current position. As a result, the defective boundary is corrected; the other boundary performs the same state as before.

First of all, we propose the simplified geometric active contour which is written as follows:

(27) $$\begin{array}{c} \frac{\partial \phi }{\partial t}=k\left|\nabla \phi \right|,\;\;\phi \left(x,0\right)=\phi_{0}, \end{array}$$

where **k** is a speed parameter which controls the speed and orientation of the evolution curve. In order to make the evolution stop on the correct boundary, **k** is defined by

(28) $$\begin{array}{c} k\left(x\right)=\left\{\begin{array}{cc} 1, & x\in \Omega_{1}\\ 0, & x\in \Omega_{3}\\ -1, & x\in \left\{\Omega_{2},\Omega_{4}\right\}. \end{array}\right. \end{array}$$

The motivation of (28) is that *k* = −1 can make the balloon force outward to expand the boundary curve, while *k* = 1 can let the force inward to shrink the boundary curve. Furthermore, *k* = 0 can keep the right boundary still and protect those points from impact and ensure the final correction result is precise.

However, without any restrictive conditions, the curve will keep expanding itself on the defective boundary all the time. And it may overpass the real lung boundary and arrives at an inappropriate position at last. Thus the curve length term and the area term are introduced by the assumption that the final evolution curve should be as smooth as possible. The introduced terms are defined by

(29) $$\begin{array}{c} E^{L}=\int_{\Omega }\delta_{\varepsilon_{1}}\left(\phi \right)\left|\nabla \phi \right|dx,\;x\in \Omega .\\ E^{A}=\int_{\Omega }H_{\varepsilon_{1}}\left(\phi \left(x\right)\right)dx,\;x\in \Omega , \end{array}$$

where *ε* is a parameter that controls the nonzero interval in the delta Dirac function *δ*_*ε*_1__(*x*). Their gradient flows are calculated by

(30) ∂ϕ∂tL=δε1ϕdiv∇ϕ∇ϕ,∂ϕ∂tA=δε1ϕ.

To maintain the smoothness of the evolution curve, the curve length term is embedded into the geometric active contour model; thus the new geometric active contour model is defined by

(31) $$\begin{array}{c} \frac{\partial \phi }{\partial t}=k\left|\nabla \phi \right|+\kappa_{1}{\frac{\partial \phi }{\partial t}}_{L}+\kappa_{2}{\frac{\partial \phi }{\partial t}}_{A}\\ \phi \left(x,0\right)=\phi_{0}, \end{array}$$

where **ϕ**_0_ denotes the final lung mask of Section 2.3 and *κ*_1_ and *κ*_2_ denote the gradient flow distribution in the proposed geometric active contour model.

In order to maintain the evolution of the level set function stable, the Gaussian convolution method which is proposed by Zhang et al. [17] is introduced into this model. The level set update function by the Gaussian kernel function is as follows:

(32) $$\begin{array}{c} \phi_{n}^{\prime }=\phi_{n-1}+\Delta t\Delta \phi ,\;\;\phi_{n}=G_{\sigma_{1}}*\phi_{n}^{\prime }, \end{array}$$

where *G*_*σ*_1__ is a Gaussian kernel function with the standard deviation of *σ*_1_, Δ*t* is the time step, and Δ*ϕ* denote the result which is obtained by the first equation of (31). Besides, *ϕ*_*n*_ serves as the initial contour for the next iteration. To the best of our knowledge, it is the first time that the geometric active model is proposed to correct the lung edge in computer-aided diagnosis technique.

**Figure 8:**
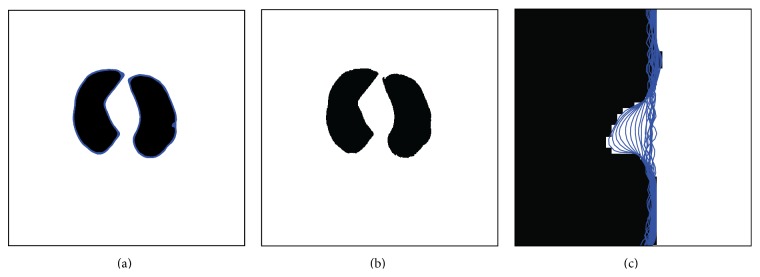
The corrected results. (a) The evolved active contour; (b) the final correction result; (c) the corrected part by the proposed method.

In the correction process, the initial contour for the geometric active contour model is set as the result of the initial lung region mask without the airway and background. Then update the level set function **ϕ** according to (32) until it arrives at a stable state. Finally, the corrected boundary, which is shown in Figure 8, is given as the zero level set of **ϕ**. In the figure, both the result of the correction method and the evolving active contour are provided. Particularly in Figure 8(c), there are all the curves in the entire correction process, which also implies the perfect effect of the proposed correction method.

## 3. Experiments

In this section, the experiments are performed on the proposed model. The experiments focus on the following aspects: (1) the superior effect of *g* ^∗^ over *g*; (2) the global minimum detecting ability of the proposed model; (3) the advantages of GLg over the state-of-the-art active contour model; (4) the correction effect analysis. The experiments are finished on a notebook computer with Intel 2.10 GHz CPU and Matlab 8.0. The parameters in the proposed model are set as follows: *α*′ = 0.9, *β*′ = 0.1, *λ* = 1 × 10^3^, *μ* = 3*λ*, *σ* = 3, *κ* = −0.05, *ω* = 1, *ε* = 1, *ε*_1_ = 0.5, *κ*_1_ = 5*n*, *κ*_2_ = 0.1, and *n* denotes the iteration times. The used 3 CT data sets are obtained from the hospital in Guangzhou, China; the rest of 25 sets are selected from the Lung Image Database Consortium (LIDC).

**Figure 9:**
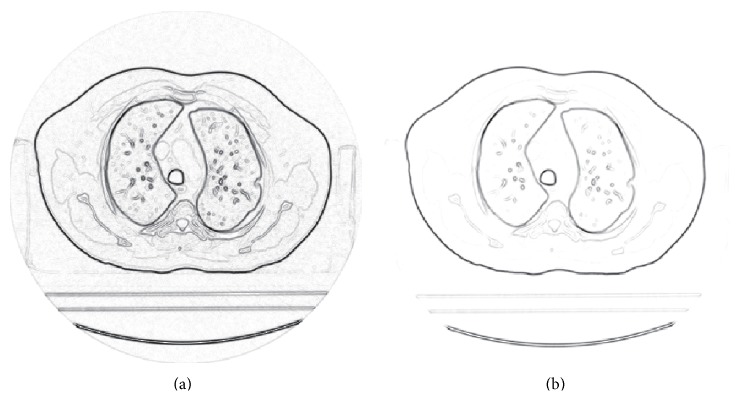
The edge detection capability of *g* and *g* ^∗^. (a) The detected edge by *g*; (b) the detected edge by *g* ^∗^.

First of all, we test the boundary detecting capability of *g* ^∗^ in the given image. In order to display the advantages of *g* ^∗^, the traditional edge detection function *g* is introduced too. The detected edges are shown in Figure 9, from which it is easy to find that *g* is affected by noise and additional background. However, due to the usage of the statistical information, *g* ^∗^ is insensitive to the noise and background and gets a clear edge-information-contained image. It is worth noting that *g* ^∗^ gets all the edges of our interesting structures except for some ribs and the spine.

**Figure 10:**
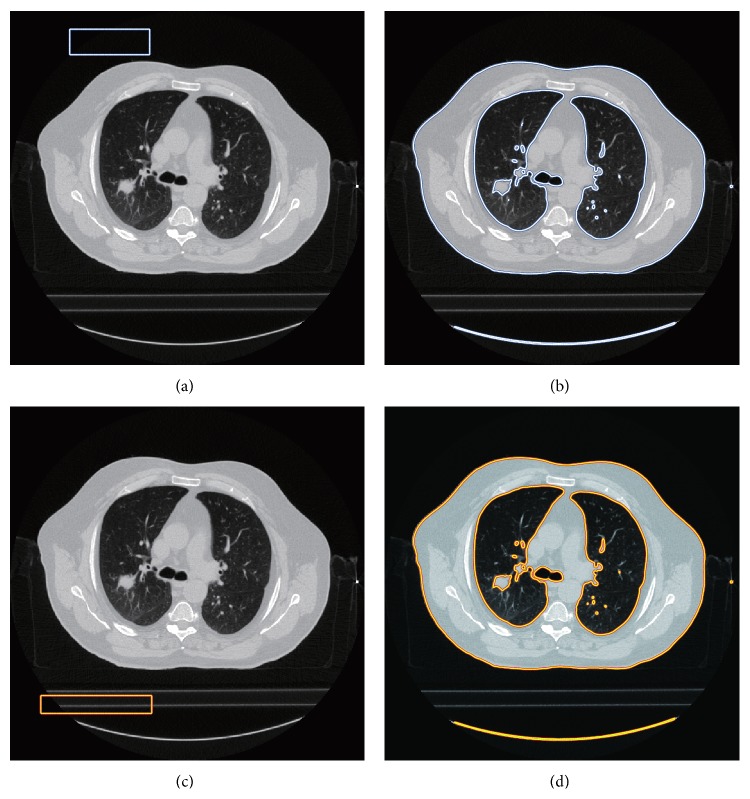
The segmentation results under two different initial contours. (a) and (c) are two initial contours; (b) and (d) are the segmentation results.

In order to demonstrate the global minimum detection capability of the proposed model, we select two different rectangle initial contours which are shown in Figures 10(a) and 10(c) correspondingly. The segmentation results are shown in Figures 10(b) and 10(d). From this experiment, it is easy to find that the proposed model gets the uniform segmentation results under two different initial contours, from which we can know that the proposed model can get the global minimum no matter what kinds of initial contour we select.

**Figure 11:**
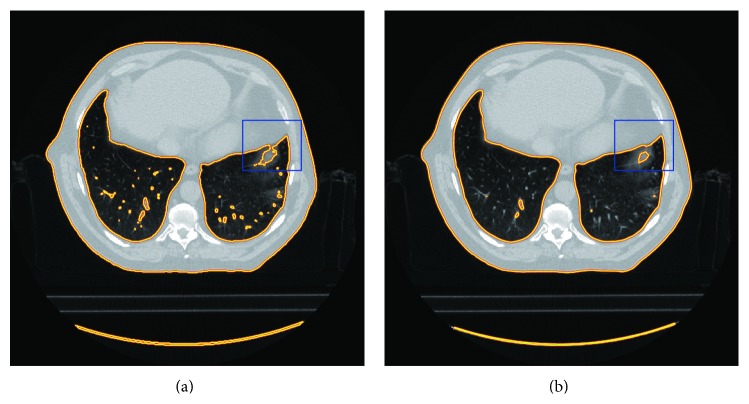
The comparison between the optimal threshold method and GLg model. (a) The segmentation result of the optimal threshold method. (b) The segmentation result of GLg model.

In the following experiment, we verify the advantages of GLg model over the popular optimal threshold method. The segmentation results are given in Figure 11, in which Figure 11(a) shows the segmentation result of the optimal threshold method and Figure 11(b) displays the segmentation result of the proposed model. From the results, it is clearly seen that the optimal threshold method fails to segment this image for there is intensity inhomogeneity in the box region. However, with the help of *g* ^∗^, GLg model detects the true boundary in the box region. Although a circular region in the box region is identified as the outer part of the lung, this region can be corrected by filling the inner hole by the morphology method; thus this part of region is not affected by the final segmentation result. Note that the hole-filling method is a common process in the previous models, and it is not our special process to deal with the segmentation result.

**Figure 12:**
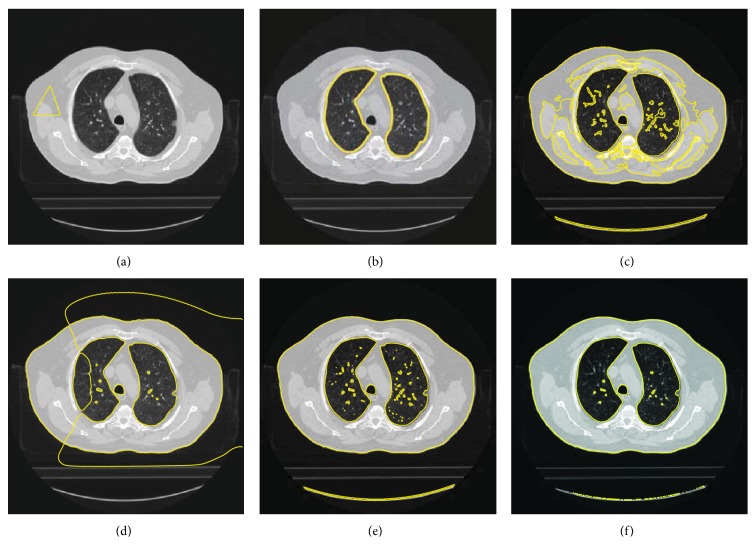
The comparison between the proposed method and other models. (a) The initial contour and the original image. (b) The segmentation result by expert. (c) The segmentation of LBF model. (d) The segmentation of LIF model. (e) The segmentation of GCLGIF model. (f) The segmentation of the proposed model.

To verify the segmentation ability of the proposed model, we compare the proposed model with some other models. The segmentation results are listed in Figure 12.  Figure 12(a) gives the initial contour and the original image, and Figure 12(b) shows the segmentation result by an expert. Figures 12(c)–12(f) provide the segmentation results of LBF model [15], LIF model [17], GCLGIF [20] model, and the proposed model correspondingly. From those figures, it is easy to find that LBF model and LIF model are sensitive to the initial contour. As combined models, GCLGIF model and the proposed model can achieve a satisfactory segmentation result. The segmentation results of the last two models approximate the segmentation result of the expert. Particularly, it is seen that the proposed model overcomes GCLGIF model in the following experiment.

For evaluating the proposed model quantitatively, another globally convex local and global intensity fitting energy (GCLGIF) model, which is also a global-and-local-combined active contour model, is introduced in this experiment. The major difference between those two models is the different edge information and different adaptive weight function. In GCLGIF model, the edge information is given by *g*; the weight function is *ω*. In GLg method, the edge information and the weight are obtained from *g* ^∗^ simultaneously which can give more useful edge information for evolving the level set function. The experiment is performed on a CT set which contains 141 CT slices; the initial active contour for both kinds of models is obtained by the threshold of −500 Hounsfield units (HUs) [5]. It is worth noting that although the proposed segmentation model can segment images accurately in every kind of initial contour which can be found in Figure 10, the above initial contour is used for reducing computational time; it does not give a better initial contour to the segmentation model. In the experiment, if we take the GCLGIF segmentation result as the golden standard, the mean segmentation accuracy of GLg method in those 141 slices achieves 99.97%. Additionally, due to the different edge information and weight function, GLg needs less time to segment all slices than GCLGIF model. Besides, the standard deviation and the coefficient of variation of all the 141 time data are also compared which are shown in Table 1. Besides, we also offer the time improved rate (TIR) in Table 1, which is defined by

(33) $$\begin{array}{c} \text{TIR}=\frac{t_{1}-t_{2}}{t_{1}}\times \%, \end{array}$$

where *t*_1_ denotes the time of the GCLGIF model and *t*_2_ denotes the time of the GLg model.

It should be noted that not only the mean time but also the standard deviation and the coefficient of variation are smaller than those of the GCLGIF model. Besides, from the TIR value, it is known that, under the nearly same accuracy, GLg model is more efficient than GCLGIF model.

**Figure 13:**
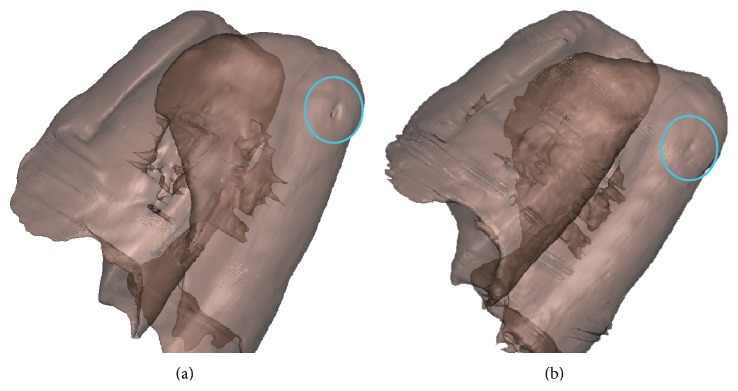
The 3D model of the corrected lung. (a) The 3D lung model before correction; (b) the 3D model after correction.

In order to display the correction effect intuitively, the 3D model of the segmented lung and the edge corrected lung is reconstructed in Figure 13. From Figure 13(a), it is easy to find that there is a hole that surrounds the juxtapleural nodule in the signed circle region. After edge correction, the hole is almost filled by the geometric active contour model, which can be seen in Figure 13(b). It is interesting to observe that below the circular region of Figure 13(b) the rib profile is more obvious after edge correction. Unfortunately, the hole is not filled perfectly; there is a tiny defect in the circular region of Figure 13(b). The leading reason is that the evolving level set function should be regularized as a signed distance function by the Gaussian convolution, which makes the zeros level set not match the real edge absolutely which can be seen in Figure 8(c). However, if we observe the tiny sag region between two ribs in Figure 13(b), the size of the unfilled hole is very small too; that is, the loss of the imperfect filling process is very slight which can be verified by Figure 8(c) too.

**Figure 14:**
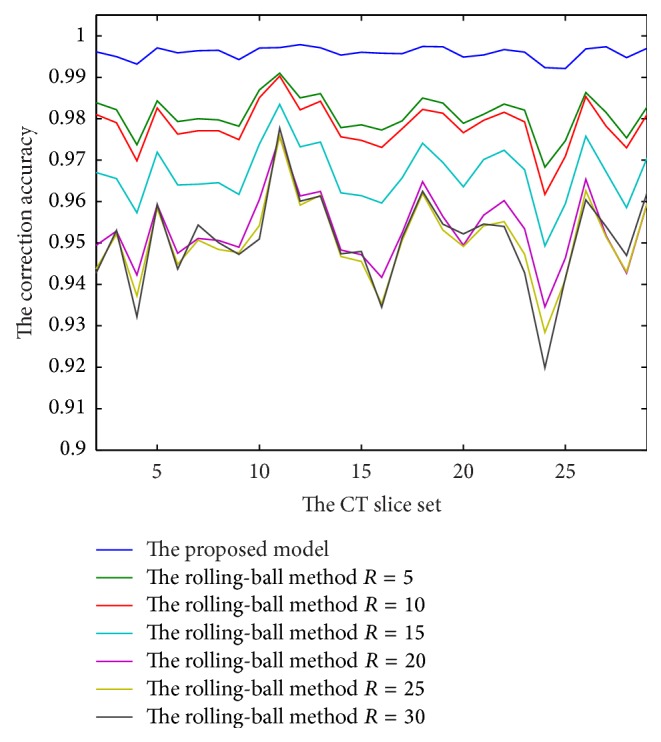
The mean correction accuracy of our method and the morphology method under 28 different CT slice sets.

Finally, in order to examine the correction accuracy of the proposed model, the correction results of GLg model are compared with the correction results by hand which are obtained under the guidance of the expert. We select 28 CT slice sets from the LIDC Database; the correction results by hand are selected as the golden standard; the mean correction accuracy of every set is shown in Figure 14. From those accuracy data, it is easy to find that the minimum of the proposed correction accuracy is above 99%; that is, they outperform their morphology-based counterparts, which implies the proposed model is a feasible model.

## 4. Conclusion

A new automated lung extraction and the edge correction method combines CV model, LBF model, the globally convex segmentation method, the split Bregman method, the fractal theory, and the geometric active contour model. The detection accuracy of the proposed new edge detection function is more accurate than the traditional edge detection function. With the help of the split Bregman method, the useful information of the new adaptive weight function, and the new edge information, the proposed segmentation model that combines the CV model and LBF model can segment the given image quickly and get the global minimum no matter where the initial contour lies. Furthermore, with the help of the fractal theory and the statistical threshold, the proposed method can detect the defective edge block successfully. In addition, the geometric active contour model can fill the hole that surrounds juxtapleural nodules automatically. Several experiments demonstrate the accuracy and efficiency of our correction model.

## Figures and Tables

**Table 1:** The computational effect comparison between *g*- and *g* ^*^-based models.

Model	$\overset{-}{t}$	Std	CoV	TIR
GCLGIF	0.37799	1.1139	2.9468	—
GLg	0.3543	0.80846	2.2818	6%

## References

[1] Bae K. T., Kim J.-S., Na Y.-H., Kim K. G., Kim J.-H. (2005). Pulmonary nodules: automated detection on ct images with morphologic matching algorithm—preliminary results. *Radiology*.

[2] Yim Y., Hong H., Beom Seo J., Kim N., Jin Chae E., Gil Shin Y. (2009). Correction of lung boundary using the gradient and intensity distribution. *Computers in Biology and Medicine*.

[3] Johnson P. T., Hahn H. K., Heath D. G., Fishman E. K. (2008). Automated multidetector row CT dataset segmentation with an interactive watershed transform (IWT) algorithm: part 2—body CT angiographic and orthopedic applications. *Journal of Digital Imaging*.

[4] Li Q., Katsuragawa S., Doi K. (2001). Computer-aided diagnostic scheme for lung nodule detection in digital chest radiographs by use of a multiple-template matching technique. *Medical Physics*.

[5] Hu S., Hoffman E. A., Reinhardt J. M. (2001). Automatic lung segmentation for accurate quantitation of volumetric X-ray CT images. *IEEE Transactions on Medical Imaging*.

[6] Avila N. A., Kelly J. A., Dwyer A. J., Johnson D. L., Jones E. C., Moss J. (2002). Lymphangioleiomyomatosis: correlation of qualitative and quantitative thin-section CT with pulmonary function tests and assessment of dependence on pleurodesis. *Radiology*.

[7] Armato S. G., Sensakovic W. F. (2004). Automated lung segmentation for thoracic CT: impact on computer-aided diagnosis. *Academic Radiology*.

[8] Sonka M., Hlavac V., Boyle R. (1999). *Image Processing, Analysis, and Machine Vision*.

[9] Itai Y., Kim H., Ishikawa S. Automatic segmentation of lung areas ba sed on snakes and extraction of abnormal areas.

[10] Silveira M., Marques J. Automatic segmentation of the lungs using multiple active contours and outlier model.

[11] Lee M., Park S., Cho W., Kim S. Medical image segmentation using a geometric active contour model based on level set method.

[12] Liu S., Li J. Automatic medical image segmentation using gradient and intensity combined level set method.

[13] Xu C., Prince J. L. (1998). Snakes, shapes, and gradient vector flow. *IEEE Transactions on Image Processing*.

[14] Chan T. F., Vese L. A. (2001). Active contours without edges. *IEEE Transactions on Image Processing*.

[15] Li C., Kao C.-Y., Gore J. C., Ding Z. (2008). Minimization of region-scalable fitting energy for image segmentation. *IEEE Transactions on Image Processing*.

[16] Wang L., He L., Mishra A., Li C. (2009). Active contours driven by local Gaussian distribution fitting energy. *Signal Processing*.

[17] Zhang K., Song H., Zhang L. (2010). Active contours driven by local image fitting energy. *Pattern Recognition*.

[18] Wang L., Li C., Sun Q., Xia D., Kao C.-Y. (2009). Active contours driven by local and global intensity fitting energy with application to brain MR image segmentation. *Computerized Medical Imaging and Graphics*.

[19] Wang X.-F., Huang D.-S., Xu H. (2010). An efficient local Chan-Vese model for image segmentation. *Pattern Recognition*.

[20] Yang Y., Wu B. (2012). Split Bregman method for minimization of improved active contour model combining local and global information dynamically. *Journal of Mathematical Analysis and Applications*.

[21] Armato S. G., Li F., Giger M. L., MacMahon H., Sone S., Doi K. (2002). Lung cancer: performance of automated lung nodule detection applied to cancers missed in a CT screening program. *Radiology*.

[22] Armato S. G., Giger M. L., Moran C. J., Blackburn J. T., Doi K., MacMahon H. (1999). Computerized detection of pulmonary nodules on CT scans. *Radiographics*.

[23] Dajnowiec M., Alirezaie J. Computer simulation for segmentation of lung nodules in CT images.

[24] Zhang X., McLennan G., Hoffman E. A., Sonka M. (2005). Automated detection of small-size pulmonary nodules based on helical CT images. *Information Processing in Medical Imaging*.

[25] Pastor L., Pousse A., Manzoni P., Kastler B. (2005). A pulmonary nodule modeling tool as a diagnostic aid for lung HRCT images. *Computerized Medical Imaging and Graphics*.

[26] Ko J. P., Betke M. (2001). Chest CT: automated nodule detection and assessment of change over time—preliminary experience. *Radiology*.

[27] Yim Y., Hong H. (2008). Correction of segmented lung boundary for inclusion of pleural nodules and pulmonary vessels in chest CT images. *Computers in Biology and Medicine*.

[28] Pu J., Roos J., Yi C. A., Napel S., Rubin G. D., Paik D. S. (2008). Adaptive border marching algorithm: automatic lung segmentation on chest CT images. *Computerized Medical Imaging and Graphics*.

[29] Weickert J. (1998). *Anisotropic Diffusion in Image Processing*.

[30] Chan T. F., Esedoglu S., Nikolova M. (2006). Algorithms for finding global minimizers of image segmentation and denoising models. *SIAM Journal on Applied Mathematics*.

[31] Goldstein T., Bresson X., Osher S. (2010). Geometric applications of the split Bregman method: segmentation and surface reconstruction. *Journal of Scientific Computing*.

[32] Foroutan-pour K., Dutilleul P., Smith D. L. (1999). Advances in the implementation of the box-counting method of fractal dimension estimation. *Applied Mathematics and Computation*.

